# Role of Leptin in Inflammation and Vice Versa

**DOI:** 10.3390/ijms21165887

**Published:** 2020-08-16

**Authors:** Antonio Pérez-Pérez, Flora Sánchez-Jiménez, Teresa Vilariño-García, Víctor Sánchez-Margalet

**Affiliations:** Department of Medical Biochemistry and Molecular Biology, and Immunology, Virgen Macarena University Hospital, University of Seville, 41009 Seville, Spain; aerolazure@gmail.com (F.S.-J.); tvgarcia@gmail.com (T.V.-G.)

**Keywords:** leptin, inflammation, obesity, leptin resistance, microbiota

## Abstract

Inflammation is an essential immune response for the maintenance of tissue homeostasis. In a general sense, acute and chronic inflammation are different types of adaptive response that are called into action when other homeostatic mechanisms are insufficient. Although considerable progress has been made in understanding the cellular and molecular events that are involved in the acute inflammatory response to infection and tissue injury, the causes and mechanisms of systemic chronic inflammation are much less known. The pathogenic capacity of this type of inflammation is puzzling and represents a common link of the multifactorial diseases, such as cardiovascular diseases and type 2 diabetes. In recent years, interest has been raised by the discovery of novel mediators of inflammation, such as microRNAs and adipokines, with different effects on target tissues. In the present review, we discuss the data emerged from research of leptin in obesity as an inflammatory mediator sustaining multifactorial diseases and how this knowledge could be instrumental in the design of leptin-based manipulation strategies to help restoration of abnormal immune responses. On the other direction, chronic inflammation, either from autoimmune or infectious diseases, or impaired microbiota (dysbiosis) may impair the leptin response inducing resistance to the weight control, and therefore it may be a cause of obesity. Thus, we are reviewing the published data regarding the role of leptin in inflammation, and the other way around, the role of inflammation on the development of leptin resistance and obesity

## 1. Introduction

Acute inflammation is a protective response that is engaged to defend and restore physiological functions and homeostasis. Acute inflammation is actually an essential part of the healing process. It starts rapidly, and symptoms may last for a short time, a few days at most. The inflammatory response can only achieve this goal by overriding or suppressing incompatible homeostatic controls. However, in its attempts to restore homeostasis, inflammation may enforce and propagate homeostatic changes, which results in chronic inflammation, a slow condition caused by a misactivation of the immune system that keeps the organism in a long-term state of high alert, which is detrimental and can result in chronic pathological states, even in the case of low-grade chronic inflammation [1,2]. In this context, pathways of systemic inflammation have been recognized as an essential component in the pathogenesis of different multifactorial diseases (type 2 diabetes and gestational diabetes, cardiovascular diseases, cancer, obesity, etc.) encompassing chronic inflammatory diseases [3,4]. Moreover, the inflammatory response observed in these pathophysiological conditions does not seem to be triggered by the classical signals of acute inflammation, infection and injury, but it appears to be supported by tissue malfunction or homeostatic imbalance. In recent years, interest has been captured by the discovery of novel mediators of inflammation, such as adipokines. Several inflammatory stimuli, such as cytokines and Toll-like receptor (TLR) ligands, induce or inhibit their expression, although this system is not fully understood. Adipokines are soluble proteins secreted by the white adipose tissue, a highly dynamic organ with a huge number of functions in physiological and metabolic processes. In fact, apart from its known roles regulating energy balance and metabolism, the white adipose tissue also modulates inflammatory and immune responses, through the secretion of adipokines. Adipokines comprise of a very heterogeneous group of mediators, some of which proinflammatory proteins, such as leptin.

Leptin, the product of the *LEP* gene, is a 16 kDa peptide hormone secreted mainly from adipose tissue, which plays an integral role in the regulation of body weight and energy expenditure [5]. Circulating leptin levels (physiological range approximately 16 ng/mL) reflect the amount of energy stored in the adipose tissue and are correlated with the degree of obesity. Thus, obese individuals typically produce higher leptin than leaner individuals [6,7,8,9]. Initially, the effects of leptin were thought to be only centrally mediated. However, leptin plays a role in a quite diverse range of physiological functions both in the central nervous system and at the periphery. The past 20 years of research on leptin have provided important insights into the intricate network that links nutrition, metabolism, reproduction as well as immune functions [7,8,9,10] and inflammation. These actions of leptin are consistent with its production by various tissues and organs, such as the stomach, skeletal muscle, pituitary cells and the placenta [6,11].

This pleiotropic nature of leptin is supported by the universal distribution of leptin receptor (LEPR), which shows structural similarity to the class I cytokine receptor family [12,13,14,15,16]. At least six alternatively spliced forms have been identified, differing in the lengths of their cytoplasmic regions, known as LEPRa, LEPRb, LEPRc, LEPRd, LEPRe and LEPRf [12,16]. The short isoform is distributed in almost all peripheral tissues and seems to mediate the transport and degradation of leptin and besides, it show distinct signaling capabilities that include the activation of mitogen-activated protein kinase (MAPK) pathway [17]. The long form isoform of LEPR (LEPRb) predominates in the hypothalamus in areas that are responsible for the secretion of neuropeptides and neurotransmitters that regulate appetite, body weight [14,15,18] and bone mass [19]. Finally, the product of the cleavage process, the so-called soluble leptin receptor, is the main binding protein for circulating leptin and modulates its bioavailability.

Leptin resistance (impaired signaling) is present in obesity, producing hyperleptinemia. Since leptin acts as a proinflammatory adipokine, the hyperleptinemia may contribute to the chronic inflammatory state of obesity. On the other hand, chronic inflammation may impair leptin action producing leptin resistance by interfering in leptin receptor signaling. The leptin resistance in the hypothalamus impairs the weight control that may lead to obesity.

In the present review, we focus on the role of leptin as a mediator of inflammation in the pathogenesis of several chronic disorders and how this knowledge could be instrumental in the design of leptin-based manipulation strategies to help restoration of abnormal responses. In addition, the role of chronic inflammation in the development of leptin resistance, which may lead to obesity is also reviewed in the present work.

## 2. Leptin Signaling in Immune Cells

The LEPR is ubiquitously expressed on the surface of immune cells both peripheral (such as monocytes/macrophages, and T and B cells) and CD34+ hematopoietic bone-marrow precursors [20,21]. Similar to other receptors of the family, LEPR lacks intrinsic tyrosine kinase activity and requires the activation of receptor associated kinases of the Janus family (JAKs). While the short-form contains only the JAK2 intracellular signaling site, the LEPRb contains an extracellular domain and an intracellular domain that bears a JAK2 signaling site, as well as three tyrosines (Tyr) that can be phosphorylated. This suggests that the binding of JAK2 is particularly important downstream of leptin.

The JAK (Janus kinases)/STAT (signal transducers and activators of transcription) pathway is one of the main signaling cascades activated by leptin in LEPRb promoting the complete activation [22,23,24]. After ligand binding, JAKs autophosphorylate and tyrosine phosphorylate various STATs. Activated STATs then dimerize and translocate to the nucleus, where specific gene responses are elicited [15,25]. Different pathways in addition to STATs are known to be involved in LEPR signaling. Thus, the mitogen-activated protein kinase (MAPK) family and the phosphatidylinositol 3-kinase (PI3K) signaling cascade become also activated by leptin, as we have previously found in peripheral blood mononuclear cells [26,27]. Therefore, the JAK2–PI3K, JAK2-Tyr 985–ERK1/2 (extracellular signal-regulated kinases or also called mitogen-activated protein kinases) and JAK2-Tyr 1138–STAT3 pathways have emerged as examples of pathways by which leptin can induce immune cell activation.

### 2.1. Leptin and Innate Immunity

The innate immune system is affected by leptin, and recent research has uncovered important mechanisms of functional regulation. Innate immune cells respond to infection and also influence the adaptive response. Leptin receptors have been found in monocytes, polymorphonuclear and natural killer (NK) cells.

#### 2.1.1. In Monocytes and Macrophages

Both the long (LEPRb) and short isoforms have been found to be expressed, if in fact, constitutive association of JAK2 and JAK3 with LEPRb has been reported, with the subsequent activation by tyrosine phosphorylation of STAT3 [28], the MAPK family and the PI3K signaling cascade [26,27,29]. It is well-established the role of leptin as a growth factor for the monocytes, promoting phagocytic function and proliferation of circulating monocytes, inducing the production of proinflammatory cytokines (TNF-α, IL-6 and IL-12) and stimulating the oxidative burst as well as the chemotactic responses mediating the inflammatory infiltrate [30,31]. On the other hand, the ROS production in HIV infected patients is an indicator of programmed cell-death in monocytes [32]. In this sense, leptin stimulation of these monocytes partially inhibited the production of ROS [33], suggesting that the antiapoptotic role of leptin may be partly mediated by the inhibition of an oxidative burst, in addition to other signaling pathways, such as MAPK in HIV-positive monocytes [27].

#### 2.1.2. Polymorphonuclear Cells

Polymorphonuclear cells have been found to express the leptin receptor in vitro and in vivo [34,35]. Particularly, in neutrophils, it has been found only in the short form of LEPR [36], which is enough to signal inside the cell through MAPK signaling pathways. In these cells, leptin seems to behave as a survival cytokine, similar to G-CSF and promotes chemotaxis [37,38] and the secretion of oxygen radicals, through direct and indirect mechanisms [34]. In eosinophils and basophils leptin also seems to be a potent activator through its positive role in chemotaxis, cytokines release and cell survival. For instance, in eosinophils, human leptin plays a key role in the host defense system against parasitic infections [39] and, thus, the level of eosinophilia might indicate the relative severity of the infection due to the invasion by the parasites [40].

#### 2.1.3. Human NK Cells 

Human NK cells constitutively express both long and short forms of LEPR. In fact, leptin signaling is necessary for normal NK cell immune function. Leptin actions in NK cells include cell maturation, differentiation, activation and cytotoxicity [41,42], as well as increased secretion of IL-12 [43]. Therefore, the main role of leptin in this context is the ability to increase immune activity and cell proliferation and to decrease the apoptotic rate of NK cells.

#### 2.1.4. Other Immune Cells

The expression of leptin and leptin receptors has also been demonstrated on mast cells, suggesting paracrine and/or autocrine immunomodulatory effects of leptin on mast cells [44]. Finally, although leptin acts as an activator, chemoattractant and survival factor (via NF-κB and PI3K-AKTsignalling), it may also be implicated in maturation and migration of dendritic cells (DCs) [45]. In this context, some studies have shown that immature DCs primed with leptin were licensed to skew the immune response toward the Th1-type and, moreover, it was also able to induce the activity of autologous CD8+ T cells in terms of perforin and IFN-γ production [46].

### 2.2. Leptin and Adaptive Immunity

Although the mechanisms of leptin regulation of the T cell function are not fully understood, leptin has also been demonstrated to modulate the adaptive immune response, which is classically divided into T helper 1 and 2 immune responses on the basis of the produced cytokine pattern. T helper 1 lymphocytes produce mainly proinflammatory cytokines that are necessary for macrophage activation and the cell-mediated response, whereas T helper 2 lymphocytes secrete modulatory and anti-inflammatory peptides that are important factors for the activation of B cells and basophils. Evidence indicates the role of the leptin in the maintenance of thymic maturation of double positive CD4+/CD8+ cells, reducing thymic apoptosis [47] as well as preventing glucocorticoids-induced apoptosis in thymocytes. In fact, chronic leptin replacement in mutant mice lacking leptin expression (ob/ob mice) restores the T-cell function, increasing the secretion of the proinflammatory cytokines. Thus, the effect of leptin polarizing T cells towards a Th1 response seems to be mediated by stimulating the synthesis of IL-2, IL-12 and IFN-ɣ and the inhibition of the production of IL-10 and IL-4 [29,30].

Besides, leptin receptor signaling in T cells is required for Th17 differentiation [48], which has a paramount role in the promotion and maintenance of inflammation and autoimmunity [49,50]. Leptin is also able to modulate the regulatory T cells (Treg) function. In this sense, leptin can act as a negative signal for the proliferation of human Treg via the mTOR pathway [51]. This supports the possibility of new antileptin-based approaches for the immunotherapy of conditions characterized by low numbers of Tregs, such as obesity, type 2 diabetes mellitus (T2D) and metabolic syndrome.

Therefore, leptin actions in T cell populations involve different processes leading to increase the immune activity by enhancing the polarization of naive T helper cells to a Th1 phenotype. Moreover, leptin increases Th17 cell proliferation while decreases Treg cell proliferation through mTOR activation.

B cells have emerged as crucial players in regulating inflammation in murine visceral adipose tissue, by presenting antigens to T cells, secreting proinflammatory cytokines, and secreting pathogenic antibodies [52], contributing to local and systemic inflammation [53]. In contrast to macrophages and T cells, little is known about the role of B cells in response to leptin. However, leptin seems to play a central role also in the modulation of B cell compartment. In fact, B cells express the long form of LEPR on the cell surface and leptin induces the secretion of proinflammatory cytokines (such as TNF and IL-6) and the anti-inflammatory and immunoregulatory cytokine IL-10 via JAK–STAT and p38MAPK–ERK1/2 signaling in B lymphocytes [54]. Moreover, leptin is necessary for B cell development and can augment the B cell population by increasing proliferation and decreasing apoptotic rate. Therefore, a role of leptin in B-cells generation and activation has been reported [55].

## 3. Leptin as a Mediator of Inflammation

### 3.1. Leptin Deficiency and Infection Diseases

Malnutrition affects around 800 million people of the world population [56]. Malnutrition and fasting are associated with nutrients insufficiency and affects both innate and acquired immunity [57,58]. That is why, people with nutrients insufficiency are vulnerable to infections because of immunosuppression [59] and defective cytokine production [60]. For example, malnutrition induces anti-inflammatory cytokines IL-4 and IL-10 and impairs proinflammatory cytokines IL-2 and IFN-γ production from CD4+ and CD8+ T cells in children. Intriguingly, the systemic leptin levels are reduced in malnutrition and in starvation, suggesting that leptin bridges a link between the nutritional status and immune system of individuals. In fact, leptin-deficiency is associated with increased susceptibility to several infections, but moreover, certain infections also caused the downregulation of systemic leptin levels and mimic a malnutrition like situation. In this context, it has been reported that a drastic fall in leptin levels during starvation increases susceptibility to lipopolysaccharide (LPS) and tumor-necrosis factor alpha (TNF-α) induced toxicity in mice. However, leptin replacement therapy markedly reverses these deleterious effects and protects the mice from fasting-induced lymphopenia [59].

Phagocytosis is a key event executed by certain immune cells to internalize the foreign pathogen inside the cell and subsequent killing. As mentioned-above, leptin induces phagocytic activity of macrophages and prevents the apoptosis of a variety of immune cells involved in both innate and adaptive immunity. In this sense, a large body of evidence has demonstrated that leptin supplementation reduced the infections of some pathogens; such as bacteria (*Listeria monocytogenes, Klebsiella pneumonia, Escherichia coli, Mycobacterium tuberculosis,* etc.…) [61,62,63,64] virus, fungus and parasite infections as well as their pathogenicity by increasing the phagocytic activity of macrophages. Even more important is the sepsis, which is a systemic inflammatory response responsible for multiple organ failure and high rate of mortality [65]. In this sense, it has also been reported that leptin replacement and leptin signaling is necessary to induce an adequate antiseptic immune response [66]. In leptin-deficient mice exogenous leptin modulated the immune response against sepsis and tremendously improved the survival rates by reducing IL-6 levels in serum and thereby controlled systemic inflammation [66]. In humans, the patients’ recovered from sepsis had higher leptin levels compared to that of non-survivors [67]. Thus, these observations reveal the neuroendocrine regulation of systemic immunity and therapeutic potential of leptin in an infectious disease [68].

The low systemic leptin levels in HIV patients [69] due to reduced adiposity might contribute to immunodeficiency [70].

As mentioned above both low systemic leptin or leptin-deficiency and impaired leptin signaling conditions are associated with increased susceptibility to infections. The impaired leptin signaling could be a cause of defective immunity due to the loss of interdisciplinary regulation among immunologic, metabolic and neuro endocrinological aspects. In this respect, LEPR mutation (Q223R) or polymorphism (rs1137101), which is a homozygous allelic mutation that results in impaired STAT3 signaling is likely to increase the susceptibility for dissemination of infection. Leptin was shown to be protective against *C. difficile* colitis by inducing STAT3 inflammatory pathway, which is impaired in the LEPR Q223R mutation [71].

Suppressor of cytokine signaling 3 (SOCS3) is a protein involved in the negative regulation of cytokines that signal through the JAK/STAT pathway including leptin receptors. SOCS3 typically inhibits T cells proliferation and activation by directly targeting CD28. This is the mechanism of viruses such as hepatitis-B, influenza, HIV, and Epstein Barr virus [72,73], which induce SOCS3 expression to ensure their survival and evade the host immunity by inhibiting IFN-α/β JAK/STAT signaling [74,75,76,77]. It has been described that a mutation (Tyr 1138 Ser) in tyrosine 1138 residue located in the intracellular domain of LEP-Rb isoform mediates STAT3/SOCS3 signaling, which results in decreased chemokine production and immune cells recruitment at the site of infection in mucosal gut tissue.

Parasite infections are reported to cause damage to intestinal mucosal epithelial cells by inducing the activation of mesenteric lymph nodes and perturbations in the adjacent adipose tissue to secrete leptin [78]. Thus, parasites may induce the malnutrition state, which is the hall mark of low systemic leptin levels [79] and disturb the host immunity. However, high serum leptin levels were reported in several parasitic infections [80], possibly due to acute inflammation and production of IL-1β, TNF-α and IL-6 caused by the gut infections [81]. This is important as leptin functions as an eosinophil survival factor in humans [39], which plays a key role in the host defense system. In addition, it promotes regeneration and intestinal integrity as well as inhibition of apoptosis in intestinal epithelium [82,83]. In fact, an integral leptin signaling via MAPK, STAT3 and AKT pathways was found to be protective against parasites in intestinal epithelial cells in response to leptin [84]. For instance, leptin was able to maintain the defense against the *L. donovani* infection through the classical activation of macrophages by inducing the phosphorylation of Erk1/2 and Akt kinase [63].

It has also been suggested that leptin might be a potential adjuvant tool in vaccination strategies as the lack of appropriate immuno-adjuvant could be one of the potential reasons for a lack of efficacy of some vaccines in preclinical studies. In this sense, leptin could restore an inflammatory response without eliciting adverse side-effects since it is produced endogenously [85,86]. However, the immunostimulatory potential of leptin cannot be neglected in vaccines development, as an adjuvant alone [87]. The co-immunization of leptin in conjugation with a vector expressing virulence have shown to be able to produce protective immunity, indicating the importance of leptin and its signaling in the generation of a host protective immune response [88].

Therefore, leptin could be a novel approach for protection against the infections in human population susceptible under certain pathological conditions such as malnutrition, diabetes mellitus or HIV infection [89,90,91,92].

### 3.2. Leptin as an Inflammatory Mediator in the Obesity-Associated Immuno-Metabolic Disorders: Diabetes, Cardiovascular Disease, Autoinmune Diseases and Cancer

**Figure 1:**
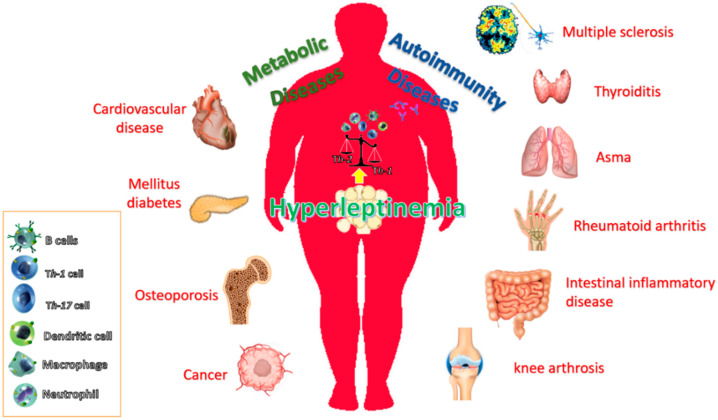
Leptin action contributes to chronic inflammation in obesity. Obesity is associated with increased leptin levels, which at the local or systemic level activate the cells of the innate and adaptive immune system. In this context, leptin can directly promote environmental conditions that in turn promote the loss of immune self-tolerance and priming immune cells for Th1 phenotype (proinflammatory). The elevated circulating leptin levels in obesity contribute to the low-grade inflammatory background, which makes obese individuals more susceptible to an increased risk of developing metabolic diseases such as cardiovascular diseases, T2D, as well as, degenerative disease including autoimmunity diseases (multiple sclerosis, thyroiditis, rheumatoid arthritis, intestinal inflammatory disease and knee arthrosis among others) and cancer.

The incidence of obesity and its associated disorders is increasing worldwide. It is known that obesity predisposes individuals to an increased risk of developing many diseases, including atherosclerosis, diabetes, certain cancers and some immune-mediated disorders [93,94,95]. This is because obesity is associated with a chronic inflammatory response, which is characterized by abnormal cytokine production, increased synthesis of acute-phase reactants, such as C-reactive protein (CRP), and the activation of proinflammatory signaling pathways [93]. Figure 1 summarizes these pathophysiological conditions associated with obesity and the possible role of leptin.

Research in the past few years has identified important pathways that link metabolism with the immune system and vice versa. Many of these interactions between the metabolic and immune systems seem to be orchestrated by a complex network of soluble mediators derived from immune cells and adipocytes. In this sense, in addition to adipocytes, which are the most abundant cell type in white adipose tissue, adipose tissue also contains preadipocytes (which are adipocytes that have not yet been loaded with lipids), endothelial cells, fibroblasts, leukocytes and, most importantly, macrophages. These macrophages are bone-marrow derived and the number of these cells present in white adipose tissue correlates directly with obesity. In fact, the adipose tissue of obese individuals also contains a large number of macrophages compared to lean individuals [96,97]. Certain cytokines such as, CC-chemokine ligand 2 (CCL2) produced by adipocytes, has recently been identified as a potential factor contributing to macrophage infiltration into adipose tissue. Once macrophages are present and active in the adipose tissue, they, together with adipocytes and other cell types present in the adipose tissue, might perpetuate a vicious cycle of macrophage recruitment and production of proinflammatory cytokines. In fact, macrophages in adipose tissue seem to be the main source of TNFα, however, adipocytes contribute almost one third of the IL-6 concentration in the circulation of patients who are obese. In addition, other products of adipose tissue such as, leptin, are thought to provide an important link between obesity, insulin resistance and related inflammatory disorders. Therefore, in obesity-related high plasma leptin conditions, inflammation would occur when signal transduction pathways was activated, such as the activation of NFκβ, by the binding of leptin to its receptor and subsequent release of the inflammation factors, for instance TNFα [98]. In this sense, the elevated ERK1/2 phosphorylation by leptin is followed by increased NFκB activation and TNFα secretion, which is in agreement with a previous report that indicated leptin has proinflammatory action, involving proinflammatory cytokines TNFα through NFκB regulation [99].

#### 3.2.1. Type 2 Diabetes Mellitus

Type 2 diabetes mellitus or non-insulin dependent diabetes mellitus is a disease of chronic hyperglycemia that leads to severe and sometimes fatal complications such as kidney failure, heart disease and death [100]. The natural history of T2D in humans leads from insulin resistance to compensatory hyperinsulinemia, and pancreatic cell dysfunction [101]. It has been reported that subclinical, low-grade inflammation might have an important role in the pathogenesis of obesity related insulin resistance and T2D [102]. Biomarkers of inflammation, such as TNF, IL-6 and CRP, are present at higher concentrations in individuals who are insulin resistant and obese, and decreased expression are observed after weight loss [103]. Therefore, the presence of these proinflammatory mediators may be biomarkers to predict the development of T2D. They might also lead to a state of vascular endothelial dysfunction and vascular inflammation, all of which promote the development of atherosclerotic cardiovascular disease. In addition, insulin resistance might be partly accelerated by an acute-phase reaction as part of the innate immune response, in which large amounts of proinflammatory mediators are released from adipose tissue. Moreover, since plasma leptin levels are positively correlated with body mass index (BMI) and obesity is a risk factor for T2D, the relationship between leptin and T2D has being extensively studied.

In searching for the mechanisms involved in inflammation-induced insulin resistance, SOCS proteins [104,105], endoplasmic-reticulum (ER) stress [106], the inhibitor of nuclear factor-κB (NF-κB) kinase-β (IKKβ) of NF-κB activation and the JUN N-terminal kinase (JNK) signaling pathways have been all associated with the development of insulin resistance. Intriguingly, activation of these pathways is regulated by leptin, the proinflammatory mediator released mainly by adipocytes that link the immune system with obesity-related insulin and leptin resistance. For instance, leptin signaling is inhibited by the overexpression of SOCS3 [107], which affects JAK/STAT pathway by binding to the phosphorylated Tyrosine-985 (pTyr985) of LEPR and induces dephosphorylation of JAK2 [108]. Protein tyrosine phosphatases (PTPs), the phosphatase and tensin homolog (PTEN), receptor-type PTPe (RPTPe) and PTP1B also induce dephosphorylation of JAK2 and inhibit leptin signaling. The expression of PTP1B and T cell PTP (TCPTP) is upregulated in a high-fat diet and obesity, and inhibits leptin-mediated STAT3 phosphorylation [109]. This is important as the PTP1B mediated ER stress induces leptin resistance [110,111], possibly by inhibiting STAT3 phosphorylation.

An activated JNK pathway by ligation of TLRs is also an important regulator of insulin resistance in mouse models of obesity. In this sense, TLRs are a family of receptors in the innate immune system that mediate signal transduction pathways through the activation of transcription factors that regulate the expression of proinflammatory cytokines in several cell types and tissues [112]. More specifically, it has been reported that TLR4, involved in modulating the innate immunity (proinflammatory macrophages) [113,114], is an important mediator of insulin resistance and inflammation through its activation both by elevated exogenous ligands (e.g., dietary fatty acids) and endogenous ligands (e.g., free fatty acids), which are elevated in obesity. Moreover, TLR4 activation also leads to increased transcription of proinflammatory genes, resulting in the elevation of cytokine, chemokine as well as reactive oxygen species and eicosanoid levels that promote further insulin-desensitization.

Taken together, several proinflammatory cytokines, SOCS proteins, ER stress, the IKKβ pathway of NF-κB activation and JNK signaling pathways are all associated with the development of insulin resistance, indicating that various proinflammatory mediators released by adipocytes, in addition to the initially described proinflammatory cytokine TNF, link the immune system with obesity-related insulin resistance.

Therefore, increased understanding of theses signaling pathways-mediated effects on insulin action present the opportunity and challenge of developing related therapeutic approaches for improving insulin sensitivity.

#### 3.2.2. Cardiovascular Diseases

It has been suggested that leptin is one of the mediators of atherosclerosis by favoring an inflammatory state that promotes the recruiting of monocytes to the arterial intima, and inducing proinflammatory cytokines [115,116]. Moreover, LEPR is present in atherosclerotic lesions, and ob/ob mice, which are leptin deficient, are protected from atherosclerosis in spite of obesity [117]. On the other hand, it is not clear whether increased leptin or leptin resistance is the mediator of atherosclerosis [118], and clinical prospective studies are needed to further clarify the role of leptin in cardiovascular disease.

#### 3.2.3. Autoimmune Diseases

The prevalence of autoimmune diseases, such as systemic lupus erythematosus (SLE), rheumatoid arthritis (RA), multiple sclerosis (MS) and type 1 diabetes mellitus (T1D), is increasing in affluent countries and associates with serum leptin levels [119]. Consistently, it has been demonstrated that leptin-deficient mice showed resistance or less susceptibility to the development autoimmune diseases [120]. Serum leptin levels are higher in RA patients with high disease activity, correlate well with disease activity and decrease significantly when disease is well controlled [121]. In fact, the leptin concentrations are significantly higher in patients with active erosive RA [122]. Even though an inverse correlation between leptin concentrations and inflammation exists in patients with active RA, plasma leptin concentrations did not significantly differ from those in healthy controls. This suggests that active chronic inflammation may lower plasma leptin concentrations.

High leptin levels are also related with a higher prevalence of other immune diseases, such as SLE [123], and also with increased susceptibility to the development of osteoarthritis (OA) [124]. In fact, it has been hypothesized that the increased predisposition of females to develop OA could be due to the higher circulating leptin levels observed in females [124] in comparison with males. Recently, leptin has been found to promote SLE by increasing autoantibody production and inhibiting immune regulation [125,126].

Obesity is also associated with other inflammatory autoimmune diseases, such as ulcerative colitis, Crohn’s disease and psoriasis [127,128], and increased leptin expression has also been reported in Behcet´s disease, psoriasis, thyroiditis and during the acute phase of ulcerative colitis [129,130,131,132]. Besides, in inflammatory bowel disease patients, systemic leptin levels are increased compared to normal healthy donors [133]. Concerning experimental autoimmune encephalomyelitis (EAE), it has been shown that ob/ob mice are resistant to the development of this model of multiple sclerosis. This resistance is abolished by the administration of leptin, which is accompanied by a switch from a Th2 to Th1 pattern of cytokine release [134]. In addition, and in concordance with these reports, it has been noticed that the onset of the disease is preceded by an increase of circulating leptin [135]. Furthermore, it has been demonstrated that acute starvation, which is accompanied by a decrease in circulating leptin levels, delays the onset of the disease and attenuates the symptoms. Recently, it has been shown that leptin levels are negatively correlated with CD4^+^ CD25^+^ regulatory T-cells during multiple sclerosis [134], suggesting that this negative association may have major implications in the pathogenesis of multiple sclerosis, as well as in the development of different autoimmune diseases characterized by Th1 auto-reactivity [134]. This interesting report indicates that leptin is produced by immune cells during acute EAE, and suggests that this hormone could be participating in the development of CNS-inflammatory diseases not only in an endocrine fashion but also by an autocrine or paracrine mechanism. In summary, regulation of leptinemia is complex and additional studies are necessary to clarify whether leptin is a real actor or a simple mediator in the inflammatory process of these autoimmune diseases.

#### 3.2.4. Cancer

Finally, increasing evidence also indicates that obesity is associated with tumor development and progression. Thus, in the context of obesity, the convergence of chronic inflammation, insulin signaling dysregulation, altered availability of lipids and other macromolecules as well as changes in adipokine signaling appear to be involved in the pathogenesis of cancer [136].

Leptin associated to the excess of adiposity influences the risk, prognosis and progression of cancer. Although the underlying mechanisms are still unclear, both leptin and its receptor expression and function have been positively correlated with cancer progression in some endocrine-related cancers [137] and this effect seems to be mainly mediated by LEPR activation of PI3K, ERK1/2 and Jak2/Stat3 signaling pathways [138,139,140,141]. These pathways regulate the expression of several cancers related genes such as cyclin D1, COX-2, VEGF and potentiates several procarcinogenic processes including angiogenesis, antiapoptosis, cell proliferation, migration and mesenchymal transformation [142,143,144]. This contributes to various steps of tumor progression, from cancer stem cell activity, survival, growth and proliferation to metastatic invasion in different types of cancer cells [145,146,147,148,149,150]. In the inflammatory context, leptin may promote molecular changes capable of modulating the behavior of tumor cells and the surrounding microenvironment, which include cancer and adipose-derived stem cells, cancer-associated adipocytes, epithelial cancer cells, fibroblasts and also immune cells. Leptin modulates both innate and adaptive immunity through its action in different cell types [151]. In this sense, leptin may contribute to the local proinflammatory mechanisms. As an example, it was shown that leptin increases IL-18 expression and secretion in TAMs, leading to increased migration and invasion of breast cancer cells [152].

In addition, leptin has a key role in the antitumor immune defense. This immunomodulatory action of leptin has been demonstrated on NK function, which is crucial for an effective antitumor response [153]. However, the exact role of leptin as a negative or positive modulator could be dependent on the dose or time effect [154,155]. Obesity has recently been found to be favorable for the response to immune checkpoint inhibitors in different tumors [156] so cytokine homeostasis, and more specifically, leptin homeostasis, could also be an important factor considered as both the modulated and modulator of the future efficacy of therapies in cancer.

A causative link between inflammation and carcinogenesis has been demonstrated. Chronic inflammation is a well-established risk factor for cancers, where genetic instability and epigenetic modification could be induced through cytokine signaling or through the generation of reactive nitrogen and oxygen species [157,158]. However, there exists a more complex crosstalk among inflammation, immune cells and cancer cells throughout the phases of elimination, equilibrium and escape in cancer immunoediting. While cancer-related inflammation confers at first the immunosuppressive activity to the tumor microenvironment (TME), it is also responsible for the epithelial-to-mesenchymal transition (EMT), tumor invasion and also the generation of a premetastatic environment in the context of immunological tolerance [159].

Adipose expansion and inflammation associated to obesity promote the cells from adipose tissue to become part of this cancer microenvironment, thus enhancing protumoral effects. Increased levels of growth factors and cytokines like leptin, decrease proinflammatory TH1 cells and increase TH2 cells and Tregs. Under these conditions, the recruitment of monocytes from the circulation leads to increased tumor-associated macrophages (TAMs) in the tumor microenvironment [157].

There is clear evidence on the association of various adipokines and obesity-related cancers [160]. In this sense, either as an independent factor or by mediating estrogens action, leptin has been proposed as a key link between obesity and different types of cancer. Thus, several data strongly support the involvement of leptin in common endocrine related cancer in women [161], especially, breast cancer [145,162,163]. Additionally, leptin have been suggested as part of the mechanisms involved in the development of obesity-related carcinogenesis in pancreatic [164], prostate [165] and colorectal cancer [166].

#### 3.2.5. Leptin as a Therapeutic Target

Even though leptin was cloned from the obesity animal model ob/ob, which has a mutated leptin gene [167] and therefore obesity may be treated with leptin administration [168], very soon obese humans were found to have increased expression of leptin in adipose tissue [169] and leptin defects are actually rare in human obesity [170]. Thus, only a few families have been identified with leptin deficiency, where leptin replacement restores the normal weight [171,172]. Another pathophysiological leptin deficient state that can benefit from leptin replacement is lipodystrophy [173,174] with good results improving glycemic control and decreasing triglyceride levels. Leptin treatment has also been found to be effective for hypothalamic amenorrhea [175].

## 4. Inflammation as a Mediator of Leptin Resistance and Obesity

Inflammation is an adaptive response that is triggered by a wide variety of physiological and pathological processes, such as infection and tissue injury, “the classic instigators” [176]. However, these are at one end of a large range of adverse conditions that induce inflammation, and they trigger the recruitment of leukocytes and plasma proteins to the affected tissue site. Once recruited, these cells can initiate many different activities, such as increasing vascularization, recruiting additional immune cells via proinflammatory signaling and initiating the phagocytosis of debris and pathogens. The mediators involved in the onset of systemic immune responses are proinflammatory and include cytokines (IL-1β, IL-6, IL-18, TNF-α and IFN-γ), transcriptional factors (e.g., NF-κB), peptides, chemokines, enzymes, lipids and coagulation factors. When the trigger of the response is successfully neutralized, immune cells shift their activity towards a pro-resolution phenotype via anti-inflammatory signaling, including lipoxins and cytokines (e.g., IL-10, IL-37 and TGF-β).

Tissue stress or malfunction similarly induces an adaptive response, which relies mainly on tissue-resident macrophages and is intermediate between the basal homeostatic state and a classic inflammatory response [176]. Therefore, although the pathological aspects of many types of inflammation are well appreciated, their physiological functions are mostly unknown.

**Figure 2:**
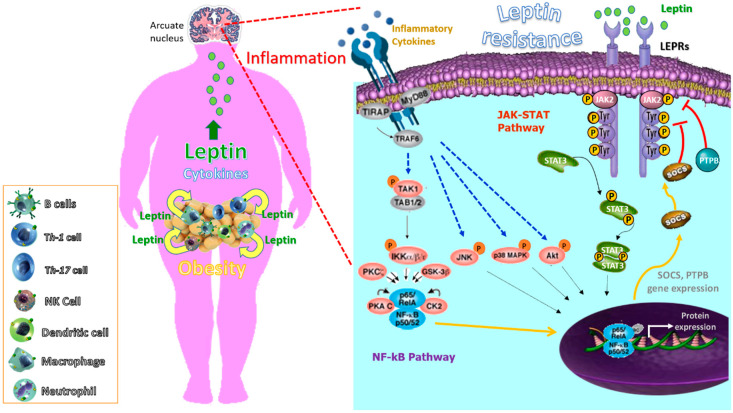
Inflammation contributes to leptin resistance in the brain at the hypothalamic arcuate nucleus, and as a result, alters food intake and energy expenditure leading to obesity. Adipocytes and central nervous system (CNS) cells interact via several secreted factors. In obesity, the adipose tissue is characterized by hypertrophy and increased infiltration of macrophages and other immune cells. The metabolic consequences of adipose tissue dysfunction are an increased synthesis of proinflammatory cytokines and adipokines, such as leptin, which impair adipocyte function. This adipose tissue dysfunction leading to chronic inflammation, not only at the local level but also at the brain level. Moreover, inflammation produced by chronic infection and autoimmune diseases contribute to leptin resistance. Recruitment and activation of NF-KB signaling molecules by proinflammatory cytokines induce SOCS3 and protein tyrosine phosphatases-1B (PTP1B), which are involved in a negative feed-back loop to block LEPR signaling via the JAK/STAT pathway and promoting leptin resistance.

One of the most intriguing aspects of studying inflammation is that the pathways of systemic inflammation have been recognized as an essential component in the pathogenesis of different multifactorial diseases encompassing chronic inflammatory rheumatic disorders, as well as a wide variety of conditions including obesity, T2D, atherosclerosis, autoimmunity and allergy [177,178]. However, these last diseases (obesity, T2D, atherosclerosis and autoimmunity allergy), different to rheumatic disorders, seem to have in common that they involve the disruption of homeostasis of one of several physiological systems that are not directly related to the host defense or tissue repair. Moreover, in these, the types of inflammatory response are likely more common but of lower magnitude than the classic inflammatory responses induced by infection or injury. Regardless the cause of the inflammatory response, its ‘purpose’ is to remove the source of the disturbance, to allow the host to adapt to the abnormal conditions and, ultimately, to restore [179,180]. In this sense, the adaptive change often provides short-term benefits; however, in a chronic phase, it can become maladaptive, as exemplified by a sustained increase in leptin levels. More specifically, a transient increase in the leptin level during acute inflammation can have a short-term benefit by helping leukocytes and other cell types during infection and tissue repair. However, sustained leptin resistance could lead to obesity [181,182], cancer and autoimmune diseases. Indeed, many chronic inflammatory diseases that are not caused by infection or injury seem to be associated with conditions that were not present during the early evolution of humans, including the continuous availability of high-calorie nutrients. More specifically, hypothalamic inflammation seems to mediate leptin resistance in these chronic inflammatory conditions [183,184] or as a consequence of a fat rich diet [185]. The role of leptin in the development, pathophysiology, acceleration or complications of many diseases as a consequence of obesity seems clear [186,187,188]. Actually, leptin has been considered a therapeutic target in autoimmune diseases using leptin antagonists [189]. We propose that the chronic inflammation in autoimmune diseases may also contribute to leptin resistance in a vicious circle, as previously hypothesized in animal models, were depletion of perforin-positive dendritic cells, which control inflammatory T cells, leads to weight gain and metabolic syndrome [190]. Figure 2 summarizes the role of inflammation in leptin resistance and obesity.

### 4.1. Infectious Diseases

#### 4.1.1. Viral Infection

In addition to inflammatory diseases some infectious diseases have been related with the development of obesity, coining the new term “Infectobesity” or the obesity of infectious origin [191]. Different viral infections have been associated with obesity, including members of Adenoviridae, Herpesviridae, phages, transmissible spongiform encephalopathies (slow virus) and hepatitides [192]. The mechanisms may include the reprogramming of host metabolism, the exchange of microbiota components, and the adaptation of host immune and metabolic system in the presence of chronic viral infection, which produces changes in cytokine and interferons that may play a role in the development of obesity [193,194]. In the other way around obesity has been found to be an important risk factor for the severity of some viral infections such as severe acute respiratory syndrome coronavirus 2 (SARS-CoV-2) [195] and leptin has also been proposed as the possible link [196].

#### 4.1.2. Bacterial Infection

Mediation of the host defense mechanisms against bacterial infection occurs by an innate immune response as the primary defense and by the adaptive immune response as the secondary defense [197,198]. One of the mechanisms of bacterial escape from host defenses is the upregulation of inhibitory molecules of cytokine signaling, especially the JAK-STAT pathway [199] such as SOCS proteins [200]. The bacterial endotoxin alone can induce adipose tissue expansion [201]. The relationship of microbes and obesity has previously been reviewed [202], and the pathways involved in microbe-induced obesity have also been summarized [203]. Therefore the infectobesity hypothesis seems to be supported by much evidence [204]. The relationship also takes place in the opposite direction. Thus, obesity by excess adiposity can increase the susceptibility to infections [202].

### 4.2. Microbiota

Interest in the role that the gut microbiota plays in disease has increased in recent years, as evidence of its importance in maintaining normal physiology. It is widely accepted that this consortium of cells provides important biological and metabolic functions that cannot be performed by our human metabolism [205]. A growing body of evidence suggests the gut microbiota participates in whole-body metabolism by affecting energy balance [206,207,208], glucose metabolism [208,209,210] and low-grade inflammation [208,210,211,212] associated with obesity and related metabolic disorders. Therefore, changes in the composition of this complex ecosystem “gut microbiota” have been associated with the development of inflammatory disorders, such as obesity. For example, it has been reported that a high-fat diet profoundly affects gut microbiota composition by reducing *Bifidobacterium* spp. and Bacteroides-related bacteria, *Eubacterium rectale–Blautia coccoides* group content [208,213], as well as, *Lactobacillus* spp. and *Roseburia* spp. [214]. In this context, TLRs could play a critical role in innate immunity by integrating signals from microbiota–host interactions (e.g., proinflammatory signals). The innate immune system detects LPS via its interaction with specific proteins that complex with TLR4 (CD14/TLR4 complex) [215]. Therefore, it can be proposed that fatty acids stimulate the innate immune system, but probably in conjunction with initial stimulation by LPS of the TLR-4/CD14 complex and subsequent TLR-2 stimulation. Moreover, both TLR5 [216] and TLR2 [217] knock out mice exhibited altered gut microbiota composition and these receptors could play a central role in the development of obesity and associated disorders.

Among the putative mechanisms linking the gut microbiota with the development of obesity, growing evidence suggests that the gut microbiota contributes to host metabolism through communication with adipose tissue, which influences the development of metabolic alterations associated with obesity. However, the exact molecular mechanisms underlying this regulation are still under investigation.

Leptin resistance is a hallmark of obesity [9] and it has been demonstrated that gut microbiota control leptin action [211]. More precisely, the altered gut microbiota composition by prebiotics improves leptin sensitivity in diet-induced obese and type 2 diabetic mice [211], suggesting the gut microbiota modulations could be a novel therapeutic target to reset leptin sensitivity during obesity.

### 4.3. The Paradox of Leptin Sensitization by Inflammatory Cytokines

Similarly to leptin or leptin receptor deficiency, leptin resistance leads to morbid obesity and interleukin-1 receptor 1 (IL1R1) deficiency and the major receptor mediating the biological function of the IL-1 cytokine family (activates inflammatory signaling pathways) also leads to a higher degree of obesity and metabolic disturbance [218,219,220]. Moreover, it has been demonstrated that LEPR and IL1R1 might physically interact [221]. In fact, IL1R1 has been identified as a mediator that increases leptin sensitization secondary to the action of celastrol, an effective drug treatment of obesity [222]. This effect of the IL1R1 in increasing leptin sensitivity is against the general dogma that cytokine/inflammatory signaling pathways have a key role in aggravation of obesity and associated metabolic diseases [1,223] and support the idea that cytokine signaling could be useful for beneficial metabolic purposes. Thus, the development of cytokine resistance could be one of the mechanisms underlying development of endoplasmic reticulum stress and obesity [222,224].

## 5. Conclusions

Inflammation is classically recognized as an essential step for the control of microbial invasion. However, now it especially represents an important process for maintenance of biological homeostasis. An aberration of these mechanisms may favor the development of various diseases, in which a relevant role is mediated by the molecular and cellular components of the innate immune system. Moreover, it is well known how the host nutritional status and metabolism can affect also the immune response. In this context, leptin, the adipose tissue-derived cytokine, has been shown to participate in a wide range of biological functions that include the activation of the immune system in the innate-adaptive frontier, underlining the link among immune function/homeostasis, metabolism and nutritional state. Thus, leptin may be one of the mediators of inflammation responsible not only in autoimmune diseases but also in other inflammatory disorders. In the opposite direction, chronic inflammatory states due to metabolic, autoimmune or infectious diseases may lead to leptin resistance at the central level, which is a known cause of obesity, therefore increasing leptin levels and further fueling the inflammation state. However, many aspects concerning leptin’s interactions with the inflammation and immune system remain unclear. Novel elements belonging to the innate immunity are continuously discovered (microRNAs, inflammasomes and the danger signals, NK cells), which synergistically enhance inflammatory responses through the integration of a multiplicity of pathways [225]. All of them have allowed us to establish unexpected links among seemingly different chronic diseases, which seems to have inflammation as the “common soil” [225]. That is why the investigation of the role of leptin in the regulation of the immune response remains a challenge for the future. The discovery of common biochemical pathways, which link metabolism and immune tolerance, could be possibly exploited to harness beneficial potential in the modulation of these pathologies.
